# Work Engagement of Employees in Moonlighting: A Moderated Mediation Model From a Boundaryless Career Perspective

**DOI:** 10.3389/fpsyg.2021.693547

**Published:** 2021-08-02

**Authors:** Zhen Peng, Qingsong Wang, Siwei Wang

**Affiliations:** International Business School, Beijing Foreign Studies University, Beijing, China

**Keywords:** boundaryless career orientations, work engagement, role conflicts, relational psychological contracts, job engagement, organizational engagement, organizational climate for openness

## Abstract

Using a panel of 324 Chinese employees in public sectors, this paper examines the work engagement of employees in moonlighting with the proxy of boundaryless career orientations. We divided work engagement into job engagement and organizational engagement and test their relation to boundaryless career orientations. The results demonstrate that boundaryless career orientations are positively related to job engagement *via* the mediating effects of role conflicts, and negatively related to organizational engagement through the mediating effects of the relational psychological contracts. Moreover, organizational climate for openness moderates the negative correlation between boundaryless career orientations and role conflicts. There is no significant evidence provided for a moderating effect of organizational climate for openness between boundaryless career orientations and relational psychological contracts.

## Introduction

In an era of increased longevity and a globalized economy, organizational boundaries became more ambiguous, and traditional organizational careers were less desirable (Arthur et al., 2005). Individuals are no longer bound to one single organization (Eby et al., 2003; Abele and Spurk, 2009), but gain sequences of experiences across different organizations and jobs (Arthur, 1994b; Eby et al., 2003; Cybal-Michalska, 2020). This has led to the emergence of a notion—a boundaryless career—characterized by transcending organizational memberships and taking a range of forms of employment beyond traditional assumptions (Peiperl et al., 2002). In this boundaryless career era, a rising population of employees begin to moonlight, that is to take on part-time jobs in addition to regular obligations of one. Holding multiple jobs in more than one organization, moonlighting employees may be provided with experience, motivation, and meaningfulness that could hardly be built in their primary jobs (Atherton et al., 2016).

However, moonlighting may also involve problems that overshadow the positive contributions of it (Jamal and Crawford, 1981). Many organizational managers apply strict rules of moonlighting, deeming that side hustle of employees stands a chance of reducing their work engagement. But the phenomenon of moonlighting is more complicated than assumed, with, still, little is known about it. Current studies on moonlighting mostly regard this behavior as a variable, focusing on its determinants (Guariglia and Kim, 2006; John and Winters, 2010; Abeyrathna, 2020) and consequences (Guariglia and Kim, 2004; Renna, 2006), yet neglect the variety of individual characteristics within the group of moonlighters. Acknowledging the importance of individual variances influencing work engagement, we regard moonlighting as a research context rather than a variable and borrow boundaryless career orientations to reflect different motives of moonlighting employees. We aim to provide a theoretical basis for in-depth understanding of engagement of moonlighting workers, taking into consideration their characteristics by proxy of boundaryless career orientations (Rothbard, 2001).

## Theoretical Background and Research Hypotheses

### Engagement of Moonlighting Employees

Kahn (1990) firstly defined work engagement as the way that “organization members control themselves to combine self and work roles” and divided it into three dimensions: cognitive engagement, psychological engagement, and emotional engagement. Saks (2019) divided engagement into two dimensions, including attention and commitment, from a psychological perspective. Schaufeli et al. (2002) suggested that work engagement is “a positive, fulfilling, and work-related state of mind that is characterized by vigor, dedication, and absorption.”

For employees who are engaged in different jobs and serve different organizations, i.e., moonlighters in this paper, their roles in assignments and organizations are separated. Accordingly, their work engagement can be divided into job engagement and organization engagement (Katz and Kahn, 2015). Drawing from role theory (Saks, 2019), work engagement is largely determined by various roles of an individual. Employees are not only “staff” in certain projects but also “group members” within the system, playing dual roles in the workplace (Saks, 2006). We, therefore, divided work engagement of employees into job engagement and organizational engagement according to the two separate roles (Saks, 2019). Job engagement is defined as “contribution and passion of employees for the job as workers,” while organizational engagement refers to “the sense of identity and social belonging to the organization when there is a close connection between the organizational interest and the career development of the employees” (Saks, 2006).

### Boundaryless Career Orientations

The concept of a boundaryless career was firstly proposed by Arthur in 1994 as “sequences of job opportunities that go beyond the boundaries of single employment settings” (Arthur, 1994b). A boundaryless career is characterized by the physical (Inkson, 2006) and the psychological (Sullivan and Arthur, 2006) willingness to cross boundaries. The physical dimension indicates organizational mobility preference to cross boundaries, associated with frequent changes of work and organizations. The psychological dimension is a boundaryless mindset (Briscoe et al., 2006) with which people are more likely to perceive the state of crossing boundaries.

Researchers asserted that, with the shifted paradigm of boundaryless careers, employees are paying more attention to the completion of tasks rather than building relationships with organizations (Pan and Zhou, 2015). When boundaryless careers have become a norm, employees trade their hard work for meaningfulness, a comfortable atmosphere, and competitive remuneration provided by the organization, as well as an elevation of their competency and employability (Sullivan, 1999). Therefore, we assume that moonlighting employees with higher boundaryless career orientations will focus more on their competency and employability, spending more job resources on completing tasks to meet job requirements, thus resulting in a higher job engagement. Despite the increase in job engagement, preference for a boundaryless career may also weaken the link between employees and an organization. Compared with those with a lower-level boundaryless career orientation, moonlighting employees who have a strong wish to cross boundaries may care less about their group identity and refuse to spare extra effort at team bonding activities with other colleagues, resulting in lower organizational engagement. Based on this, we put forward the following hypotheses:

Hypothesis 1a: Boundaryless career orientations are positively related to job engagement in the context of moonlighting.
Hypothesis 1b: Boundaryless career orientations are negatively related to organizational engagement in the context of moonlighting.

### Mediating Effect of Role Conflicts

Role conflicts refer to the inherent contradictions that an individual encounters in different social roles (Obermaier and Koch, 2015). Studies have shown that individuals holding additional position besides regular work may be vulnerable to role conflicts caused by role differences (Fröhlich et al., 2013). The most salient role conflicts for moonlighting employees lie between the effort spent on moonlighting jobs and that on regular works. The imbalance between the needs of the primary job and the part-time job will add to fatigue and stress, wear out emotional resources of employees, resulting in their resistance to the job and a decrease of job engagement.

These conflicts brought by multiple roles, however, may be averted by boundaryless career orientations. On the one hand, a higher boundaryless career orientation is characterized by the capability to perceive the state of crossing boundaries (Briscoe et al., 2006). A good perception of the state may enable employees to actively adjust their behaviors and cognitions, thus prevent several problems from happening. On the other hand, a higher boundaryless career orientation may provide employees with more job resources to achieve win–win performance in each position. As suggested in the previous section, employees with a strong orientation to cross boundaries are paying more attention to the completion of tasks rather than building relationships with organizations (Pan and Zhou, 2015). They may experience more psychological success with higher-level knowing-how and knowing-why competencies, career autonomy, and lowered career insecurity (Colakoglu, 2011). Moreover, boundaryless career orientations can be regarded as resources for employees from the perspective of Resource Preservation Theory (Hobfoll and Lilly, 1993), which may modify perceptions of employees of stress and tiredness in the process of crossing the boundary between primary work and moonlighting employment. When role conflicts are weak, employees will have the ability to allocate resources appropriately. Moreover, they may even utilize the resources obtained from moonlighting employment, such as new skills and networks, to better perform the tasks in primary positions.

As pieces of research have proved, role conflicts may affect job engagement of employees (Arthur, 1994a). Since role conflicts may also be weakened by boundaryless career orientations, we regard role conflicts as an important mediator in connecting boundaryless career orientations and job engagement. The higher the tendency to cross boundaries, the fewer conflicts between multiple roles are perceived, the higher job engagement will be witnessed. Based on the above reasoning, the following hypotheses are proposed:

Hypothesis 2a: Boundaryless career orientations are negatively related to role conflicts in the context of moonlighting.
Hypothesis 2b: Role conflicts are negatively related to job engagement in the context of moonlighting.
Hypothesis 2c: Role conflicts have a mediating effect between boundaryless career orientations and job engagement in the context of moonlighting.

### Mediating Effects of Relational Psychological Contracts

Psychological contracts are belief of one in the exchange arrangement between employees and their organizations (Rousseau, 1995). It is a balance between employee contribution and organizational demands; also a balance between organizational opportunity and employee desire. Psychological contracts have two common patterns, namely, relational psychological contracts and transactional psychological contracts (Robinson et al., 1994). Relational psychological contracts, formed based on long-term social and emotional cohesiveness (e.g., loyalty, support, etc.), are positively related to perceived trust of employees and a sense of belonging to an organization. It is commonly known that there must be a certain incentive for employees to devote their time and effort to organizations (Mowday et al., 1979), and relational psychological contracts may level up identification of members, bringing in more intrinsic motives to their work (Gupta et al., 2012). In parallel, transactional psychological contracts are based on economic exchange without much emotional involvement, thereby the employees perceive more extrinsic motivations, such as remunerations, rewards, promotions, etc., from the organization (Rousseau, 1990).

From the perspective of Social Exchange Theory (Cropanzano and Mitchell, 2005), the exchange arrangement between employees and their organizations has shifted in this era of boundaryless careers, so does the psychological contracts. The psychological contract, as a psychological variable, is subjective to individual characteristics of employees. Studies have shown that boundaryless career orientations are negatively related to organizational commitments (Briscoe and Finkelstein, 2009), while relational psychological contracts are positively related to organizational commitment (McInnis et al., 2009), thereby we assume relational psychological contracts between organizations and employees are negatively affected by boundaryless career orientations.

As pieces of research approved, psychological contracts are interactive with work engagement (Soares and Mosquera, 2019). If we look at them from the perspective of relational psychological contracts, psychological contracts are positively related to perceived trust and their sense of belonging to the organization (Gupta et al., 2012). Since employees with relational psychological contracts are more concerned about long-term social cohesiveness than material returns, they may have a higher organizational engagement.

Since relational psychological contracts are negatively influenced by boundaryless career orientations and positively related to organizational engagements, we assume a mediating effect of relational psychological contracts between boundaryless career orientations and organizational engagement. Based on the above, the present paper puts forward the following hypotheses:

Hypothesis 3a: Boundaryless career orientations are negatively related to relational psychological contracts in the context of moonlighting.
Hypothesis 3b: Relational psychological contracts are positively related to organizational engagement in the context of moonlighting.
Hypothesis 3c: Relational psychological contracts mediate between boundaryless career orientations and organizational engagement in the context of moonlighting.

### Moderating Effect of Organizational Climate for Openness

The concept of an organizational climate for openness was first proposed by Lewis (1936) as an organizational reaction to the external environment, which could influence human behaviors to some extent. Drawing on recent work in organizational psychology (Hofhuis et al., 2016; Brimbal et al., 2020; Carlucci et al., 2020), this present study introduces the concept of organizational climate for openness in the context of moonlighting to examine the relation between boundaryless career orientations and work engagement. Organizational climate for openness in moonlighting represents the inherent inclusiveness for moonlighting activities in an organization. It is formed by organizational cultures, disciplines, and rules, exerting an imperceptible influence on attitudes of employees toward moonlighting activities. Studies have shown that a good organizational climate can relieve stress and tiredness, helping organizational members to maintain positive attitudes toward works. From the perspective of Job Demands-Resources Theory (Bakker and Demerouti, 2017), we argue that, when there is a good organizational climate for openness, employees may have a higher degree of autonomy in the workplace, coordinating their material and spiritual resources to meet the job requirements, thereby alleviate the role conflicts and other pressures caused by moonlighting role conflicts. That is, with the strengthening of organizational climate for openness, the negative effect of boundaryless career orientations on role conflicts may be averted.

**Figure 1:**
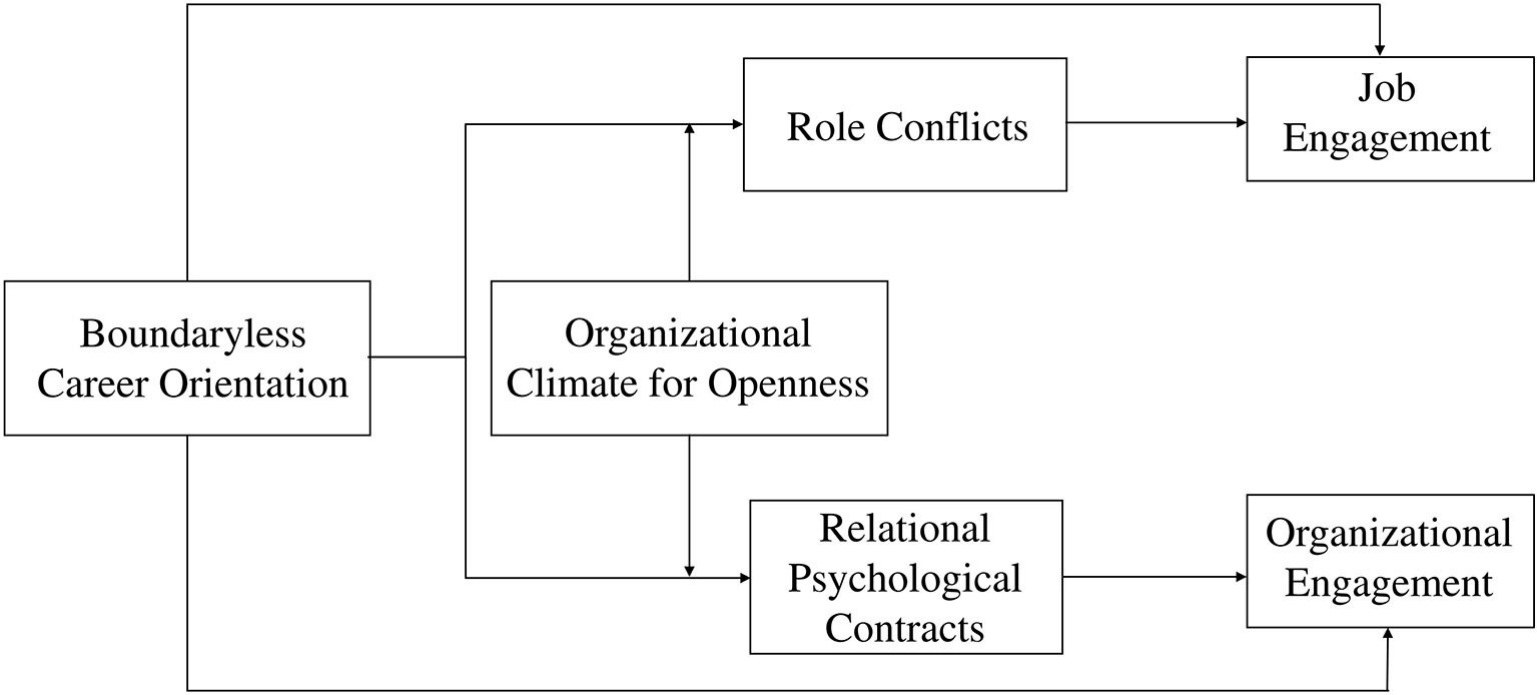
Analytic model.

When moonlighting employees have strong orientations to a boundaryless career, their relation with the primary organization, i.e., public institutions in this study, will be weakened. However, if they are provided with a more open working environment, they will perceive more trust and support from the primary organization, in return show more loyalty and motivation to work. Consequently, the negative effect of the boundaryless career orientations on the relational psychological contracts may be weakened by an organizational climate for openness. Based on the above, this article puts forward the following hypotheses:

Hypothesis 4a: The organizational climate for openness enhances the negative relation between boundaryless career orientations and role conflicts in the context of moonlighting.
Hypothesis 4b: The organizational climate for openness reduces the negative relation between boundaryless career orientations and relational psychological contracts in the context of moonlighting. Based on the above analysis, the research model in this article is shown in Figure 1.

## Materials and Methods

### Participants

The participants of this study were recruited *via* a third-party moonlighting platform. The sample included 324 Chinese employees moonlighting in addition to their regular work in the public sectors (universities, research institutes, medical institutions, etc.). The response rate is 83.3%. As this study concerns more about the individual variances, the sample size is adequate. During the data collection, each participant was invited to fill out six questionnaires, including scales of boundaryless career orientations, role conflicts, relational psychological contracts, job engagement, organizational engagement, and organizational climate for openness.

Half of the participants were women (46%), and the majority were between 31 and 40 years old (53.6%), with 35.2% under 30 and 11.2% above 41. The age ranged from 27 to 65. Besides, more than half of the participants hold a master's degree or higher (87.2%). Half of the participants reported their moonlighting jobs were not similar to their regular work (45.2%), whereas 54.8% reported their moonlighting jobs were similar to their regular work. Over the past 3 years, a majority of the participants had been engaged in moonlighting jobs for 6–12 months (76.4%), 16.4% work for 1–2 years, and 7.2% work for more than 2 years. Two-thirds of the participants spent 4–8 h weekly in moonlighting jobs (62.4%), whereas the remaining moonlighted more than 9 h every week.

Informed consent was used and each participant received 11 yuan as benefit of participating in this study. We have added the related information in the new manuscript. Before filling in the questionnaire, participants have been addressed about the difference between the “job engagement” and “organizational engagement” and knew clearly about the difference. Both “job engagement” and “organizational engagement” mentioned in this study are referred to work engagement for their original full-time employment.

### Measures

The measurement system of this study consists of six subscales, including boundaryless career orientations scale, role conflicts scale, relational psychological contracts scale, work engagement scale, organizational engagement scale, and organizational climate for openness scale.

#### Boundaryless Career Orientations Scale

We borrowed the boundaryless career orientations scale (Briscoe et al., 2006) to measure boundaryless career orientations. During the pilot study, we found out that several items in the original version were not suitable in the context of China; thus, we reduced 13 items to 8 items, measuring the boundaryless mindset (e.g., “I prefer jobs that will allow me to learn new things”) and mobility preference (e.g., “My ideal career is to work for one organization only”).

#### Role Conflicts Scale

The wide recognition of role conflicts has led the researchers to develop scales for its measurement. We adopted role conflict and ambiguity scale of House et al. (1983) because it is most suitable for our research in the context of moonlighting. We drew on the role conflict and ambiguity scale (House et al., 1983) to examine role conflicts in moonlighters, and it includes six items, such as “I often get myself involved in situations in which there are conflicting requirements” and “My authority matches the responsibilities assigned to me.”

#### Relational Psychological Contracts Scale

Our relational psychological contracts scale draws on the research of Grimmer and Oddy (2007), including seven items, such as “I am looking forward to promotion in my organization through long-term work and continuous effort” and “I am willing to work for this organization for my lifetime.” These items combined assess the content of the psychological contract.

#### Job Engagement Scale and Organizational Engagement Scale

Work engagement scale draws on research results of Saks (2019), which is an updated model of Saks (2006) model. He designed two six-item scales to measure job engagement and organizational engagement.

The updated scale, measuring job engagement, includes six items, such as “I'm passionate about my job” and “I often do extra work in my position.” The organizational engagement scale includes six items, such as “I'd love to introduce my organization (including products, services, features, strengths, etc.) to my friends” and “I'll be actively participating in the various activities of my organization.”

#### Organizational Climate for Openness Scale

Organizational climate for openness scale draws on research results on Wang and Chang (2017), which consists of four items, such as “I have autonomy in control of my work” and “My organization often provides employees with opportunities for external communication and cooperation.”

Control variables include: (1) gender; (2) age; (3) education; (4) similarity between moonlighting jobs and regular work; (5) cumulative moonlighting, working time during past 3 years; (6) weekly moonlighting hours.

All scales except control variables are measured by the Likert five-point scale, with five choices that start at one end with “strongly agree” and end at the other with “strongly disagree,” with less extreme choices in the middle three points.

### Common Method Bias

Because the measurement of all variables in this study came from the same participant, common method bias may occur. We performed Harman's single-factor test to test common error variance. If the total variance extracted by one factor exceeds 50%, common method bias is present. The results show that the total variance extracted by one factor is less than 40%, rejecting the possibility of common method bias in our measures of boundaryless career orientations, role conflicts, relational psychological contracts, work engagement, organizational engagement, and organizational climate for openness. As a result, we conclude that the data used in this study do not have a common method bias and are suitable for empirical analyses.

### Reliability and Validity

Before statistical analysis, factor analysis and reliability tests were carried out on the subscales to measure the internal consistency of the results. The results reveal that KMO values of all six subscales are greater than 0.7, suitable for factor analysis. The reliability coefficients of all variables are greater than 0.7, also indicating a good reliability. Factor loading of each variable indicator is greater than 0.5, indicating that the questionnaire has good structural efficiency. Besides, all of the six measuring scales are borrowed from valid pieces of research, thus confirm the validity of our measurements.

## Results

### Descriptive Statistical Analysis

**Figure 2:**
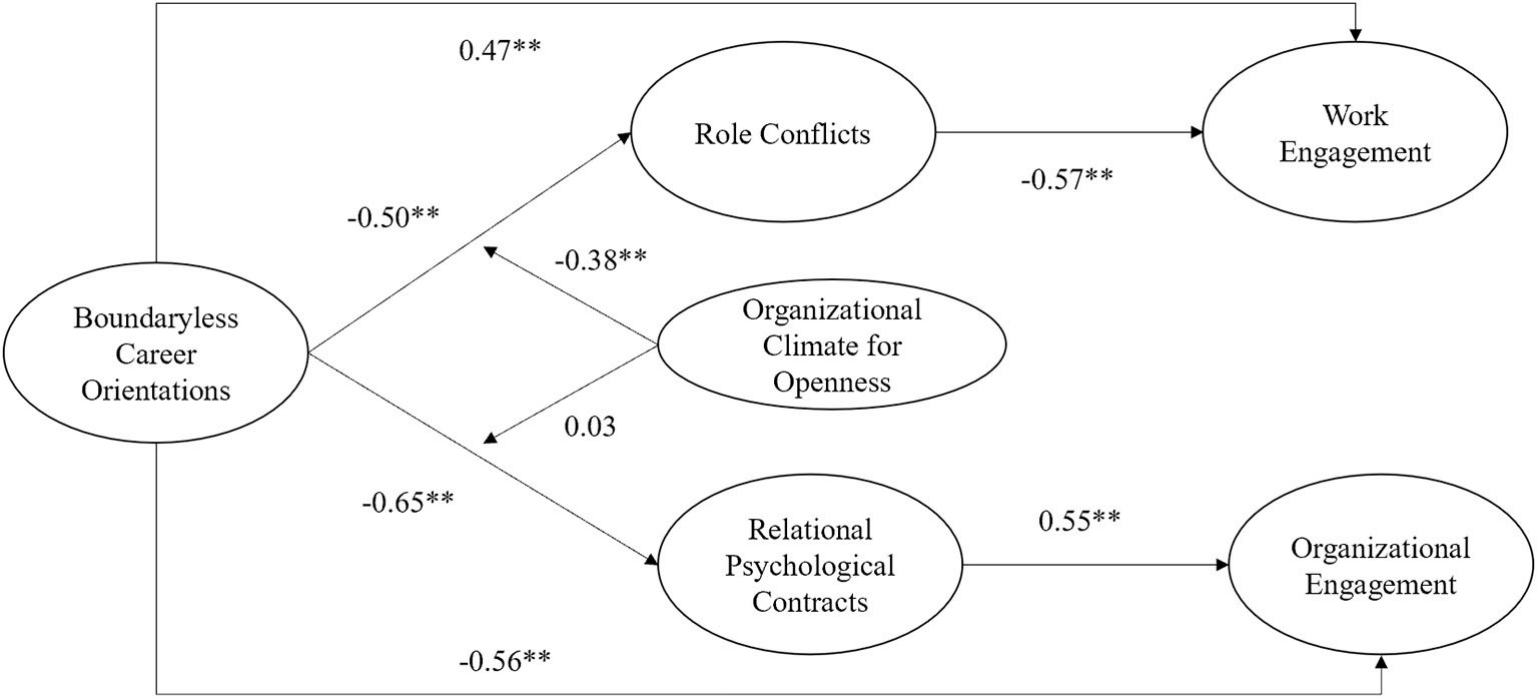
Results. ^*^p < 0.05; ^**^p < 0.01; ^***^p < 0.001.

Descriptive statistics and Pearson correlations for the construct used in the analysis are shown in Table 1. The means, standard deviations, and correlation coefficients are reported in Table 1. Figure 2 demonstrates the results and different relationships. The results show negative and significant correlation between the boundaryless career orientations and the role conflict (*r* = −*0.426; p* < *0.01*), relational psychological contracts (*r* = −*0.592; p* < *0.01*). We also found a negative and significant correlation between role conflicts and job engagement (*r* = −*0.447; p* < *0.01*). Relational psychological contracts and organizational engagement are significantly positively related (*r* = *0.578; p* < *0.01*). These results primarily support our hypotheses.

**Table 1:** Means, standard deviations, and correlations.

**Variable**	***M***	***SD***	**1**	**2**	**3**	**4**	**5**	**6**
1. Boundaryless career orientations	1.780	0.510	1					
2. Role conflicts	3.560	0.656	−0.426^**^	1				
3. Relational psychological contracts	4.074	0.646	−0.592^**^	0.323^**^	1			
4. Organizational climate for openness	4.090	0.750	−0.075	0.322^**^	0.084	1		
5. Work engagement	2.960	0.839	0.311^**^	−0.447^**^	−0.129^*^	−0.148^*^	1	
6. Organizational engagement	4.206	0.630	−0.547^**^	0.290^**^	0.578^**^	−0.014	−0.209^**^	1

* *p < 0.05*;

** *p < 0.01*;

***p < 0.001.

### Main Effect

The results of the main effect analysis can be found in Table 2. From model 1, the regression coefficient between boundaryless career orientations and job engagement is positive and significant (*r* = *0.465, p* < *0.01*), thereby verifying hypothesis 1a. From model 2, the regression coefficient of boundaryless career orientations and organizational engagement is negative and significant (*r* = −*0.575, p* < *0.01*), supporting hypothesis 1b. From Models 3 and 4, the regression coefficient is negative and significant, indicating a direct negative effect of boundaryless career orientations on relational psychological contracts and role conflicts (*r* = −*0.500, p* < *0.01*), thus validating hypotheses 2a and 3a. From model 5, the role conflicts regression coefficient is negative and significant, indicating a direct negative effect on job engagement (*r* = −*0.566, p* < *0.01*), thus validating hypothesis 2b. From model 6, the relational psychological contracts regression coefficient is positive and significant (*r* = *0.550, p* < *0.01*), indicating a direct positive effect on organizational engagement, supporting hypothesis 3b.

**Table 2:** Regression analysis (A).

**Variables**		**Model 1**	**Model 2**	**Model 3**	**Model 4**	**Model 5**	**Model 6**
		**Job engagement**	**Organizational engagement**	**Role conflicts**	**Relational psychological contracts**	**Job engagement**	**Organizational engagement**
Control variables	Gender	0.060	−0.027	0.002	−0.074	0.074	−0.002
	Age	0.127	0.049	−0.116	0.018	0.040	0.063
	Diplomacy	0.053	−0.029	−0.117	−0.062	−0.022	−0.048
	Similarity	0.024	−0.012	−0.004	0.014	0.017	−0.015
	Duration	−0.024	0.052	0.036	−0.010	−0.008	0.063
	Weekly	−0.095	0.069	0.060	0.057	−0.070	0.048
Independent variables	Boundaryless career orientations	0.465^**^	−0.575^**^	−0.500^**^	−0.650^**^		
	Role conflicts					−0.566^**^	
	Relational psychological contracts						0.550^**^
	Δ*R*^2^	0.088	0.289	0.186	0.344	0.183	0.328
	*F*-value	4.439^**^	15.492^**^	9.106^**^	19.65^**^	8.979^**^	18.37^**^

*p < 0.05;

** *p < 0.01*;

***p < 0.001.

### Mediating Effect

Mediating effect is tested by the Baron and Kenny (1986) method. Firstly, we tested the main effect of boundaryless career orientations on job engagement and organizational engagement. Secondly, the effects of boundaryless career orientations on role conflicts and relational psychological contracts as mediators were tested. Thirdly, the mediating effects of role conflict and relational psychological contract were tested.

Following steps of Baron and Kenny, the first and second steps testing the mediating effect of role conflicts are present in model 1 and model 3, while the results of the third step can be seen in model 7. The regression coefficient between role conflicts and job engagement is negative (*r* = −*0.480, p* < *0.01*), indicating a significant mediating effect. Consequently, the variable of boundaryless career orientations has an indirect positive effect on job engagement through role conflicts, that is, stronger boundaryless career orientations will reduce role conflicts, and lowered role conflicts will make work engagement stronger, verifying hypothesis 2c.

As for the mediating effect of relational psychological contracts, the first and second steps of the analysis results can be seen in model 2 and model 4, while the third step is shown in model 8. The regression coefficient between relational psychological contracts and organizational engagement is positive and significant (*r* = *0.378, p* < *0.01*), indicating a strong mediating effect. Consequently, boundaryless career orientations have an indirect negative effect on organizational engagement through the relational psychological contract, that is, stronger boundaryless career orientations will be reduced by relational psychological contracts, while lower relational psychological contracts reduce organizational engagement, validating hypothesis 3c.

### Moderating Effect

Moderating effect is also tested by the Baron and Kenny method (Baron and Kenny, 1986). The moderating effect of organizational climate for openness can be found in Table 3. Correlations with control variables are shown in Table 4. From model 9, the product term coefficient of the boundaryless career orientations and the organizational climate for openness is negative and significant (*r* = −*0.376, p* < *0.01*) and Δ*R*^2^ is 30.7%. This indicates the relationship between the boundaryless career orientations and the role conflicts is moderated by the organizational climate for openness, enhancing their negative main effect. That is to say, a stronger organizational climate for openness will enhance the negative effects of the boundaryless career orientations on the role conflicts, thus verifying hypothesis 4a. From model 10, the product term coefficient of boundaryless career orientations and organizational climate for openness is positive but not significant, meaning organizational climate for openness cannot moderate the negative relationship between boundaryless career orientations and relational psychological contracts; thus, hypothesis 4b is not supported. One possible explanation is that relational psychological contracts of employees with the organization are vulnerable to external interruptions when they are in an open climate with less difficulty encountered in crossing boundaries. Commitment of employees to an organization based on social exchange may hardly defend those interruptions; thus, organizational climate for openness may not lessen the negative relation between boundaryless career orientations and relational psychological contracts.

**Table 3:** Regression analysis (B).

**Variables**		**Model 7**	**Model 8**	**Model 9**	**Model 10**
		**Job engagement**	**Organizational engagement**	**Role conflicts**	**Relational psychological contracts**
Control variables	Gender	0.061	0.001	0.026	−0.063
	Age	0.071	0.042	−0.104	0.021
	Diplomacy	−0.003	−0.069	−0.097	−0.065
	Similarity	0.022	−0.017	0.021	0.019
	Duration	−0.007	0.056	0.010	0.015
	Weekly	−0.066	0.047	0.029	0.049
Independent variables	Boundaryless career orientations (BCO)	0.226^*^	−0.329^**^	0.959^**^	−0.593
	Role conflicts	−0.480^**^			
	Relational Psychological Contracts		0.378^**^		
	Organizational Climate for Openness (OCO)			0.951^**^	−0.095
	BCO × OCO			−0.376^**^	0.032
	Δ*R*^2^	0.199	0.386	0.307	0.274
	*F*-value	8.749^**^	20.529^**^	12.028^**^	15.292^**^

* *p < 0.05*;

** *p < 0.01*;

***p < 0.001.

**Table 4:** Correlations with control variables.

	**Gender**	**Age**	**Diplomacy**	**Similarity**	**Duration**	**Weekly**	**Boundaryless career orientations**	**Role conflicts**	**Relational psychological contracts**	**Job engagement**	**Organizational engagement**	**Organizational climate for openness**
Gender	1											
Age	−0.12	1										
Diplomacy	−0.01	−0.14^*^	1									
Similarity	−0.07	0.1	−0.18^**^	1								
Duration	−0.02	0.12	−0.02	0.24^**^	1							
Weekly	−0.04	−0.06	0.04	0.21^**^	0.27^**^	1						
Boundaryless career orientations	0.09	−0.12	−0.04	−0.07	−0.07	−0.07	1					
Role conflicts	−0.02	−0.04	−0.08	0.05	0.06	0.09	−0.43^**^	1				
Relational psychological contracts	−0.12	0.12	−0.05	0.09	0.05	0.09	−0.59^**^	0.32^**^	1			
Job engagement	0.05	0.05	0.01	−0.01	−0.04	−0.09	0.31^**^	−0.45^**^	−0.13^*^	1		
Organizational engagement	−0.07	0.13^*^	−0.08	0.06	0.1	0.1	−0.55^**^	0.29^**^	0.58^**^	−0.21^**^	1	
Organizational climate for openness	−0.05	−0.01	0.02	−0.06	0.13^*^	0.04	−0.08	0.32^**^	0.08	−0.15^*^	−0.01	1

* *p < 0.05*;

** *p < 0.01*;

***p < 0.001.

## Discussion

### Conclusions

Current studies on moonlighting mostly regard it as a variable, focusing on its determinants and consequences (Renna, 2006; John and Winters, 2010; Abeyrathna, 2020) but neglect the individual characteristics within the group of moonlighters. To fill this gap, we borrowed boundaryless career orientations as the proxy of individual characteristics to investigate its influence on work engagement of moonlighting employees. Aiming to gain an in-depth understanding of engagement of moonlighting workers, taking into consideration their characteristics, we have achieved the following conclusions:

(1) When employees hold moonlighting jobs, their boundaryless career orientations will affect work engagement in their primary work. To illustrate this influence, we divided work engagement into two different patterns, namely the job engagement and organizational engagement. This empirical study shows that among those who have strong boundaryless career orientations, their job engagement will be relatively higher and their performance of duties will remain the same or even better. In contrast, their organizational engagement may be lower, which may be attributed to their mentality beyond boundaries.
(2) Besides the direct effects on work engagement, as described above, boundaryless career orientations also indirectly influence work engagement *via* role conflicts and the relational psychological contracts. On the one hand, boundaryless career orientations facilitate job engagement by weakening role conflicts; on the other hand, high-level boundaryless career orientations reduce organizational engagement by weakening the relational organizational contracts between the organizations and their employees.
(3) Among the moonlighters that we have examined, we found out that the organizational climate for the openness in their primary workplaces will moderate the relationship between their boundaryless career orientations and role conflicts, yet its moderating effects between boundaryless career orientations and relational psychological contracts are not significant. Although the organizational climate for openness, as a resource offered by regular workplaces (Kaya et al., 2010), could help employees deal with the negative effects of role conflicts and enhance the negative impact of boundaryless career orientation on role conflict, it can hardly weaken the negative effects of the boundaryless career orientations on the relational psychological contracts.

### Theoretical Contributions

(1) As about the concept of work engagement, it remains unclear how many dimensions it comprises. Plenty of previous studies analyzed work engagement from the dimensions of vigor, dedication, and absorption (Kulikowski, 2019). This study, however, divides work engagement into job engagement and organizational engagement, taking into consideration the dual roles of employees in moonlighting. We found out that they are differently affected by boundaryless career orientations. Consequently, this is a new perspective for the investigation of work engagement among employees holding more than one position.
(2) This study clarifies the key mediating variables between boundaryless career orientations and work engagement among moonlighters and elaborates the interaction mechanism between them. Although existing studies on moonlighting behaviors have examined the variable of role conflicts as its consequences (Fröhlich et al., 2013), yet few of them have considered in-depth the link between role conflicts and work engagement. Moreover, this study has also found that relational psychological contracts play an important mediating role between boundaryless career orientations and organizational engagement of employees from the view of organizational roles of the employees.
(3) This study innovatively introduces a new variable, i.e., organizational climate for openness to explore the effect of boundary openness on work engagement. This is not only a good addition to the study of organizational climate for openness but also contributes to the theoretical construction for further understanding of the relation between boundaryless career orientations and work engagement. Since very few studies have considered the relationship between organizational climate for openness and boundaryless career orientations, role conflicts, and relational psychological contracts of employees, this study is also believed to have provided a new situational variable for the study of the work engagement among moonlighting employees and to have further illustrated the interaction mechanism between boundaryless career orientations and work engagement.

### Management Indications

This empirical study explores the influence of boundaryless career orientations on work engagement of employees in the context of moonlighting and also examines the moderating effect of organizational situational variables. Study results may shed light on management practices from the following perspectives:

(1) From the perspective of a job role, boundaryless career orientations will increase job engagement of employees, providing theoretical support for the acceptance of moonlighting behaviors. When it is widely believed wealth does not necessarily mean career success (Cutcher et al., 2019), our research proves that individual boundaryless career orientations may increase subjective well-being and feelings of success, which might contribute to positive organizational psychology both theoretically and practically. For employees having moonlighting jobs, boundaryless career orientations may facilitate them to break through organizational boundaries both physically and psychologically. On the one hand, they will make effective use of their own resources to a larger scale by cognitive surplus sharing; on the other hand, boundaryless career orientations may help employees to perceive more meaningfulness and value in work, achieving more subjective feeling of career success.
(2) From the perspective of the organizational role, the boundaryless career orientations of employees in the context of moonlighting will lead to less organizational engagement in the absence of policy intervention. This is because employees with strong boundaryless career orientations tend to neglect the importance of relational psychological contracts with an organization. They work multiple jobs and frequently travel between different organizations, thus lack time and effort to participate in activities held by the original organization with little concern about the goals of the organization. Moreover, these employees may be easily tempted by other industries and organizations, resulting in implicit loss of talents (Cutcher et al., 2019), threatening the organizational sustainable development. To benefit organizational development in the long run, managers should care more about the career development of employees, provide them with more opportunities for training and promotion so that they can perceive more recognition, care, and respect from the group, and be willing to establish long-term emotional ties and relational psychological contracts with the organization (Cutcher et al., 2019). This would eliminate the negative impact of boundaryless career orientations on organizational engagement.
(3) With the change of organizational environment and the innovation of technologies, traditional relationship between organization and employee is no longer stable (Parry and Battista, 2019), strengthening boundaryless career orientations of employees. In response to this shift of traditional careers, this paper believes that organizations should provide an open climate to increase work engagement of employees. Besides, organizations should innovate management methods to create a good organizational atmosphere for employees. Previous studies examined the mediating effect of organizational climate between human resources input and outcomes (al Damoe et al., 2017), and proved a good organizational climate will benefit human resources practices. If we take a look at the current management practices, however, it is not uncommon to find organizations adopting mandatory management strategies with complicated regulations and operational procedures, limiting the autonomy and creativity of employees who would like to take on moonlighting jobs. This will not only increase the perceived role conflicts of employees but also leave them with negative attitudes toward the organization. When the trend toward boundaryless careers seems inevitable (Cybal-Michalska, 2015), organizational managers should avoid mandatorily bonding employees with the organization by administrative means. Instead, by creating an open and autonomous organizational environment and encouraging employees to share moonlighting experiences within the organization, managers will create more flexibility and autonomy in the work environment, thus improve the job engagement of the employees.

### Limitations

Limitations and some suggestions for future directions can be identified. (1) The data in this study are collected from the self-reporting of employees in public sectors who moonlight in addition to their regular work. The limited sample size can hardly draw more general conclusions. Future pieces of research need to expand the scope and number of samples. (2) The mediating variables of this study are role conflicts and relational psychological contracts. However, the impact of boundaryless career orientations on work engagement may also be influenced by other factors, such as job transition (Forrier et al., 2015; Johnson and Matthes, 2018) and organizational commitment (Eliyana et al., 2019; Suharto et al., 2019). Further studies should consider alternative ways to include more variables. (3) We use organizational climate for openness as a situational variable to measure the engagement of employees. Future researchers may choose other variables to testify this result. (4) This study used cross-sectional data. Despite the fact that we have provided correlated data that can be used to draw conclusions, we were not able to demonstrate the causal relationship between variables, given the equal weight of different data points. In the future, the potential growth model can be used to further demonstrate the relationship between the boundaryless career orientation and the engagement of employees.

## Data Availability Statement

The raw data supporting the conclusions of this article will be made available by the authors, without undue reservation.

## Ethics Statement

Ethical review and approval was not required for the study on human participants in accordance with the local legislation and institutional requirements. The patients/participants provided their written informed consent to participate in this study. Written informed consent was obtained from the individual(s) for the publication of any potentially identifiable images or data included in this article.

## Author Contributions

ZP: substantial contributions to the conception or design of the work, final approval of the version to be published, and agreement to be accountable for all aspects of the work in ensuring that questions related to the accuracy or integrity of any part of the work are appropriately investigated and resolved. SW: acquisition, analysis, and interpretation of data for the work. QW: drafting the work critically for important intellectual content. All authors contributed to the article and approved the submitted version.

## Conflict of Interest

The authors declare that the research was conducted in the absence of any commercial or financial relationships that could be construed as a potential conflict of interest.

## Publisher's Note

All claims expressed in this article are solely those of the authors and do not necessarily represent those of their affiliated organizations, or those of the publisher, the editors and the reviewers. Any product that may be evaluated in this article, or claim that may be made by its manufacturer, is not guaranteed or endorsed by the publisher.
